# A cross-level study of the relationship between ethical leadership and employee constructive deviance: Effects of moral self-efficacy and psychological safety climate

**DOI:** 10.3389/fpsyg.2022.964787

**Published:** 2022-09-15

**Authors:** Luming Shang, Lei Yang

**Affiliations:** College of Economics and Management, Shandong University of Science and Technology, Qingdao, China

**Keywords:** constructive deviance, ethical leadership, psychological safety climate, moral self-efficacy, cross-level effect

## Abstract

Constructive deviance describes acts that benefit the organization by deviating from outdated organizational norms. Despite emerging interest in this behavior, questions remain about why and how constructive deviance occurs. This paper integrates social learning and uncertainty reduction theories, and develops a multilevel model linking team-level ethical leadership to employee constructive deviance. Surveying 313 subordinates and 52 supervisors from 15 different companies in eastern China, we find that team-level ethical leadership has a positive impact on employee constructive deviance, and that both psychological safety climate and employee moral self-efficacy partially mediate this relationship. In addition, we find a positive cross-level moderating effect of psychological safety climate. These findings contribute to understanding employees’ constructive deviance in the workplace, and provide valuable implications for managerial practices.

## Introduction

In 2006, Xiaochuan Wang, then vice president of the Chinese internet services company Sohu, decided to develop Sogou Explorer, in violation of the chairman’s decision and organizational procedures; Sogou Explorer was officially launched in 2009, and in the following years contributed nearly half of Sohu’s annual revenue. This case exemplifies, Vadera et al.’s (2013) finding that employees sometimes break organizational norms and by doing so may bring unexpected benefits to the organization. In academic circles, this behavior is defined as constructive deviance, which refers to voluntary actions whereby an employee departs from norms or procedures of an organization in the interests of organizational wellbeing (Galperin, 2012), and has been found to be beneficial for promoting individuals’ innovation performance and achieving positive changes for the organization (Mainemelis, 2010; Dahling and Gutworth, 2017). However, because it challenges the status quo and the organizational leader’s authority, constructive deviance may also have negative consequences for employees’ career development. Thus, some researchers stress that constructive deviance is an ethical decision of employees, and use a behavioral ethics perspective to explain why employees are willing to risk sacrificing their personal interests to engage in constructive deviance (Jetten and Hornsey, 2014; Zhang et al., 2021). Although these studies provide a different and interesting perspective on the emergence of employees’ constructive deviance, existing research using this perspective to explain the formation of employees’ constructive deviance is limited and requires further development. Our study addresses this deficiency and enriches the literature on constructive deviance by introducing a behavioral ethics perspective.

The crucial role of leadership style in influencing employees’ ethical decisions and moral conduct is long-established in the behavioral ethics literature (Treviño et al., 2006). Following this perspective, Zhang et al. (2021) suggest that leader moral humility can foster employees’ constructive deviant behavior by shaping their moral identity. Likewise, the literature on antecedents of employees’ constructive deviance identifies leadership style as a key influential factor (Mertens and Recker, 2020; Li and Wang, 2021; Zhou and Qian, 2021). For instance, Mertens and Recker (2020) found that empowering leadership makes employees believe that they can bravely engage in constructive deviance. However, whether and how ethical leadership affects constructive deviance remains unexplored, even though constructive deviance has moral relevance. Most Chinese employees in China, affected by Confucian moral concepts, believe that ideal ethical leaders adhere relentlessly to inner moral standards and that they influence subordinates through their own moral charisma (Yuan et al., 2022). Distinguished from other types of leadership, the essence of ethical leadership is its unique contribution to influencing employees’ ethical decision-making and fostering their ethical conduct (Brown and Treviño, 2006; Afsar and Shahjehan, 2018). Considering constructive deviance is an ethical decision, we suggest that ethical leadership may be a key predictor of employees’ constructive deviance.

Social learning theory suggests that employees learn how to behave by observing their leaders’ behaviors. Ethical leaders are moral persons who possess admired virtues and high moral character (Brown et al., 2005; Treviño et al., 2006). Applying social learning theory, previous studies note that ethical leaders can improve employees’ moral self-efficacy because employees’ efficacy expectation and outcome expectation are influenced by observational learning of moral characters from their ethical leaders, and consequently their moral self-efficacy is strengthened (Manz and Sims, 1981; Bandura, 1997; Wang et al., 2018). Employees with high moral self-efficacy believe they can convert moral beliefs into actions (Hannah and Avolio, 2010), and therefore may seek to change current inappropriate organizational procedures (Kim and Vandenberghe, 2020), which may promote constructive deviance. We thus propose that moral self-efficacy is a possible mediating mechanism linking team-level ethical leadership and constructive deviance. Furthermore, drawing on uncertainty reduction theory, employees are more likely to embrace the uncertainty and risks associated with challenging the organizational status quo in a climate of psychological safety (Edmondson and Lei, 2014; Tu et al., 2018). Therefore, we argue that such a climate may exert a cross-level mediating effect between ethical leadership and employees’ constructive deviance. Moreover, team climate is an important situational work context that can have a strong influence on employees’ attitudes and behaviors (Salancik and Pfeffer, 1978). Therefore, the extent to which team members’ moral self-efficacy influences constructive deviance may be context specific. A psychologically safe team climate provides team members with a supportive and trustworthy environment to interact with others (Kahn, 1990), which may promote translation of their moral self-efficacy into concrete actions, such as constructive deviance. In contrast, in an insecure climate with lower psychological safety, individuals may avoid risky behaviors and suppress their real opinions. Given that constructive deviance is regarded as risky, we propose that team psychological safety climate moderates the association between employee moral self-efficacy and constructive deviance.

**FIGURE 1:**
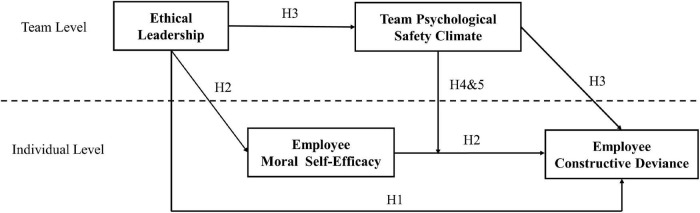
Theoretical model.

Our research makes several contributions to the constructive deviance literature. First, this is the first empirical study to examine the relationship between team-level ethical leadership and employees’ constructive deviance in the Chinese organizational context. Thus, our study provides empirical evidence on constructive deviance among employees within Chinese organizations and enriches existing theoretical understanding on the antecedents of constructive deviance. Second, most prior studies focus mainly on single-level mechanisms linking leadership style and constructive deviance, and few studies investigate multi-level mechanisms in this relationship. The present study fills this knowledge gap by introducing team psychological safety climate and employee moral self-efficacy as mediating mechanisms in our cross-level processing model. Finally, this study examines the moderating role of team psychological safety climate in the relationship between employee moral self-efficacy and constructive deviance, which contributes to a more complete picture of why and when team-level ethical leadership affects employees’ constructive deviance. The theoretical model of our study is shown in Figure 1.

## Theoretical analysis and hypothesis inference

### Constructive deviance

Galperin (2012) defines constructive deviance as a voluntary behavior that violates significant norms with the intent of improving the wellbeing of an organization, its members, or both. For example, employees are usually required to follow certain organizational rules and standards when interacting with customers, but sometimes must deviate from these established processes or standards to adequately respond to customer needs. Previous studies have stressed that constructive deviance is characterized by the following. (a) Deviations from the norms of the reference group. This characteristic reflects the differences between constructive deviance and organizational citizenship behaviors, because the latter do not involve rules-violating and risk-taking (Vadera et al., 2013). (b) Benefits to the reference group. As Galperin (2012) notes, distinguished from the construct of destructive deviance, the intent of constructive deviance is to benefit the organization. (c) Conformation to hypernorms which are “globally held beliefs and values” (Vadera et al., 2013). Constructive deviance conforms to moral norms and standards (Vadera et al., 2013), and so is theoretically different from unethical pro-organizational behavior. Although both constructs are pro-organizational, unethical pro-organizational behavior is morally problematic because it violates core societal values or norms and potentially damages the organization’s sustainability (Bryant and Merritt, 2021).

### Ethical leadership and employees’ constructive deviance

Based on social learning theory, Brown et al. (2005) propose the concept of ethical leadership, defined as “the demonstration of normatively appropriate conduct through personal actions and interpersonal relationships, and the promotion of such conduct to followers through two-way communication, reinforcement, and decision-making” (Brown et al., 2005, p. 120). As Brown et al. (2005) highlight, two crucial dimensions of ethical leadership are being a moral person and a moral manager. The former refers to the leaders’ desirable personal characteristics such as integrity, altruism, and trustworthiness. The latter refers to how a leader utilizes moral managerial measures to influence followers’ ethical decision-making and guide their ethical behaviors. Researchers on behavioral ethics have built on this work and demonstrated the positive influence of ethical leadership on many outcomes related to employees’ ethics, such as moral voice (Lee et al., 2017), knowledge sharing (Bavik et al., 2018), and internal whistleblowing (Cheng et al., 2019).

Constructive deviance—deviating from organizational norms but conforming to moral norms and standards (Warren, 2003) is increasingly regarded as an ethical decision by employees (Monin et al., 2008; Jetten and Hornsey, 2014; Vardaman et al., 2014; Zhang et al., 2021). Because ethical leadership is important in influencing employees’ ethical decision-making, we posit that ethical leadership may positively affect employees’ constructive deviance. First, ethical leaders value organizational wellbeing and speak out publicly against inappropriate organizational norms and actions that harm the interests of the organization and its members (Walumbwa and Schaubroeck, 2009; Islam et al., 2021). From a social learning perspective (Bandura, 1977, 1986), employees proactively learn and emulate their leaders’ behaviors. Consequently, employees may learn their ethical leaders’ behaviors and challenge inappropriate organizational rules, which triggers constructive deviance.

Second, at its core, ethical leadership is about contributing to the organization and positively helping others (organizational members and other stakeholders), without expecting personal gain or reward (Kanungo, 2009). Social learning theory holds that employees observe and learn their supervisors’ behavioral norms to understand how to behave at work (Bandura, 1977, 1986). As a result, ethical leaders can shape employees’ moral altruistic attitudes through the process of social learning. Such moral altruistic attitudes enhance employees’ concerns for the organization, thus, these employees will not only seek to satisfy their personal interests but also strive to benefit their organization, which may entail constructive deviance.

Finally, ethical leaders show genuine concern and care for their followers, and fully respect their interests (Brown et al., 2005). When employees feel respected and supported by their leaders, they exhibit more favorable attitudes and reciprocate with pro-organizational behaviors (Qian et al., 2017). Accordingly, constructive deviance may arise from employees’ willingness to make extra effort beyond their ordinary work duties to realize the organizational goals. In line with this reasoning, we hypothesize that ethical leadership promotes the emergence of employees’ constructive deviance.

**Hypothesis 1:** Ethical leadership is positively associated with employee constructive deviance.

### Mediating role of moral self-efficacy

Self-efficacy refers to an individual’s perception of their capability to accomplish tasks (Bandura, 1997). Hannah and Avolio (2010) expand this construct to the domain of behavioral ethics and defined moral self-efficacy as “an individual’s belief in his or her capabilities to organize and mobilize the motivation, cognitive resources, means, and courses of action needed to attain moral performance, within a given moral domain, while persisting in the face of moral adversity” (Hannah and Avolio, 2010, p. 297). They further describe moral self-efficacy as “state-like vs. trait-based,” and thus it is context-dependents. Ethical leadership can shape employees’ moral self-efficacy through the social learning processes. Social learning theory states that individuals look to their leaders’ behaviors for cues to form or develop their own attitudes and behaviors (Bandura, 1977, 1986). By observing and learning ethical leaders’ values and norms concerning moral issues, employees not only gain meaningful experience relevant to solving moral issues (Kim and Vandenberghe, 2020) but also enhance their moral capabilities (Ruedy and Schweitzer, 2011). Such moral capabilities help to increase employees’ moral self-efficacy because they develop “potential response repertoires” toward moral issues at work (Hannah and Avolio, 2010).

A high sense of moral self-efficacy implies that employees believe they can implement ethical behaviors successfully in accordance with their ethical beliefs (Ogunfowora et al., 2021). Although they may experience adversity in performing moral behaviors, employees with moral self-efficacy will remain resilient to adversity, and attempt to utilize relevant resources to overcome obstacles (May et al., 2013; Afsar and Shahjehan, 2018). Previous studies have also shown that moral self-efficacy promotes such ethical risk-taking behaviors as moral voice (Lee et al., 2017; Afsar and Shahjehan, 2018) and team organizational citizenship behaviors (Kim and Vandenberghe, 2020). Given that constructive deviance involves moral relevance and risk-taking, we argue that employees with moral self-efficacy are more likely to persist when resolving ethical dilemmas and make ethical decisions, which inspires constructive deviance. Thus, we hypothesize:

**Hypothesis 2:** Moral self-efficacy mediates the relationship between ethical leadership and constructive deviance.

### Mediating role of team psychological safety climate

Uncertainty reduction theory suggests that uncertainty is an adverse experience; therefore, individuals rely on information from their work environment to mitigate concerns and anxiety brought about by uncertainty (Lind and Bos, 2002). A psychological safety climate is a supportive environment state characterized by “the shared belief among team members that the team is safe for interpersonal risk taking” (Edmondson, 1999, p. 354). Edmondson (1999) stresses that a psychologically safe climate is a work climate characterized by interpersonal trust and mutual respect. As such, a psychological safety climate suppresses distracting interpersonal concerns and relieves team members’ fear of uncertainty. Because constructive deviance is associated with uncertainty and risk, employees may have a higher intention to conduct constructive deviance when the team climate reduces uncertainty. Drawing on uncertainty reduction theory, we expect that ethical leadership positively predicts employees’ constructive deviance by providing a psychological safety climate.

According to uncertainty reduction theory, leaders are essential in helping employees cope with uncertainty (Lind and Bos, 2002; Tu et al., 2018). Ethical leaders are moral managers who set ethical standards and utilize reward and punishment mechanisms to uniformly enforce these standards in the work context (Brown and Treviño, 2006). In this way, ethical leaders’ ethical managerial measures convey to their followers what constitutes appropriate behavior and thus clarify followers’ behavioral roles. By doing so, ethical leaders reduce subordinates’ feelings of uncertainty and increase their psychological safety. Moreover, ethical leaders care for the interests of employees, provide them fair treatment, and use ethical values to integrate teams (Brown et al., 2005). Therefore, ethical leaders promote interpersonal trust and mutual respect among members, thereby creating a psychologically safe team climate. In a psychologically safe environment, team members believe that they share mutual concern and respect with their coworkers (Edmondson, 1999). Thus, members of psychologically safe teams focus on constructive problem-solving without fear of being excluded or humiliated by other members (Bradley et al., 2012; Liang et al., 2012). Furthermore, in a team climate of strong psychological safety, members feel safe taking risks, thus, spurring their moral risk-taking behaviors. Indeed, extensive research supports the relationship between psychological safety climate and employees’ moral risk-taking behavior. For instance, Walumbwa and Schaubroeck (2009) suggest that members working in psychologically safe teams willingly engage in constructive voice behavior. Wadei et al. (2021) also find that in psychologically safe teams, members are likely to challenge the status quo. On this basis, we argue that psychologically safe climates promote employees’ constructive deviance. We thus hypothesize:

**Hypothesis 3:** Psychological safety climate mediates the positive relationship between ethical leadership and constructive deviance.

### Moderating role of team psychological safety climate

Prior studies suggest that people who work in a low team psychological safety climate exhibit worse task performance than people in a high team psychological safety climate (Edmondson, 1999; Koopmann et al., 2016). When in teams with weaker psychological safety climate, team members consider the potential risks of engaging in constructive deviance because they focus on self-protection and risk prevention. For instance, employees may be hesitant to conduct constructive deviance because they are anxious about ostracism from their coworkers or criticism for breaking organizational rules. Thus, team members in a weak psychological safety climate may regard constructive deviance as an irrational behavior with high risk and low return; even if they feel a high sense of moral self-efficacy, they may avoid constructive deviance to reduce the risks of uncertainty. In contrast, a stronger psychological safety climate usually indicates beneficial interpersonal interactions (Koopmann et al., 2016). In such a climate, members are likely to trust their coworkers and not be embarrassed to express themselves (Zhang et al., 2010), which reduces their concerns regarding constructive deviance. Therefore, we argue that a stronger psychological safety climate may increase the effect of employees’ moral self-efficacy on constructive deviance. Thus, we hypothesize:

**Hypothesis 4**: Psychological safety climate moderates the positive relationship between employee moral self-efficacy and constructive deviance. That is, the positive relationship is stronger for stronger psychological safety climates.

### Moderated mediation effect

Based on the above conjecture, we predict that team psychological safety climate moderates the mediating effect of moral self-efficacy. Specifically, we predict that the association between ethical leadership on employees’ moral self-efficacy is stronger for higher levels of psychological safety climate, which improves moral self-efficacy and so reinforces team members’ beliefs that they are capable of enacting constructive deviance. In contrast, in a climate with a lower level of psychological safety, the association between ethical leadership and employees’ moral self-efficacy may be weaker, which may lead to lower confidence in their ability to enact constructive deviance, reducing their enactment of constructive deviance. Therefore, we hypothesize:

**Hypothesis 5:** The indirect effect of ethical leadership on constructive deviance through employee moral self-efficacy is moderated by psychological safety climate such that the indirect effect is stronger when the psychological safety climate is strong.

## Materials and methods

### Procedure and samples

We issued questionnaires to 69 supervisors and 405 of their direct subordinates who engage in R&D (research and development) or customer services employed by 15 different companies in high technology, finance, and service industries in eastern China. These participants were chosen for two reasons. First, R&D and customer service jobs are flexible and members of such teams are more likely to solve problems creatively. Second, R&D and customer service jobs require members to collaborate, implying that these jobs have a high degree of task correlation, and so individual variables will generally have small between-group differences, thus making systemic between-group differences are more prominent, facilitating cross-level analysis. Before distributing questionnaires, the anonymity of responses and the definition and examples of constructive deviance were communicated to participants. To ensure that supervisor-subordinate data are well matched, we sought participants’ permission to use the initials of each subordinate’s name and the last 4 digits of their employee identification number to code the questionnaires (e.g., LN6890, etc.).

To alleviate possible common method bias, we adopted the methods recommended by Podsakoff et al. (2003) to collect survey data at two different times. At time 1, all participants were asked to respond to measures of demographic variables, and 405 subordinate employees were asked to complete questionnaires on ethical leadership and psychological safety climate in a separate survey. Four weeks later (time 2), 69 team supervisors were asked to rate their direct subordinates’ moral self-efficacy and constructive deviance. After excluding incomplete questionnaires, a total of 313 valid matched supervisor-subordinate dyads were obtained in 52 teams from 15 companies, including 52 supervisor surveys and 313 subordinate surveys, giving an overall response rate of 77.3%. Of the 52 supervisors, 44 were male (84.62%) and 8 were female (15.38%); 67.9% were in the age group 41–50 and 15.1% were above 50 years old; the overall level of education was high, with 32% having a graduate degree. Of the 313 subordinates, 62.5% were female; 44.7% were in the age group 31–40; and the average tenure was 8.89 years.

### Measures

Questionnaire items used a five-point Likert scale (1 = strongly disagree to 5 = strongly agree). To ensure clarity and consistency of the survey instrument in the Chinese context, a back-translation process was applied to the survey, as recommended by Brislin (1986). All questionnaire items are presented in the Appendix.

### Ethical leadership

A ten-item scale (Brown et al., 2005) was used to measure ethical leadership. One sample item is “Our team leader disciplines employees who violate ethical standards” *Cronbach’s alpha* was 0.933. Following previous studies (Bai et al., 2017; Christensen-Salem et al., 2020), we conceptualize ethical leadership at the team level. The results of data aggregation testing show that the values for r_wg_ and intraclass correlation index ICC (1) and ICC (2) are 0.954, 0.746, and 0.945, respectively. All three values are above the recommended thresholds (Schneider et al., 1998), indicating that aggregation to the team level is justified.

### Moral self-efficacy

A five-item scale developed by Hannah and Avolio (2010) was used to assess moral self-efficacy. One sample item is “My subordinate can fight against people who use unethical behavior to solve problems.” Cronbach’s alpha was 0.908.

### Team psychological safety climate

Liang et al.’s (2012) five-item scale was used to assess psychological safety climate. One sample item is “In my work unit, I can freely express my thoughts.” *Cronbach*′s*alpha* was 0.854. This scale indicated *r*_wg_, ICC (1) and ICC (2) values of 0.905, 0.321 and 0.739, respectively, thus supporting team-level aggregation.

### Employee constructive deviance

A 10-item scale developed by Galperin (2012) was used to measure constructive deviance. Sample items are “My subordinate sought to bend or break the rules in order to perform your job” and “My subordinate departed from dysfunctional organizational policies or procedures to solve a problem.” *Cronbach*′s*alpha* was 0.872.

### Control variables

Individual-level control variables include employees’ gender, age, and tenure were controlled, because these variables potentially impact constructive deviance. Previous studies suggest that male employees are more likely than females to exhibit constructive deviance (Dahling and Gutworth, 2017). Age and tenure may also influence constructive deviance, because employees who have a longer-term relationship with their leader (reflected in age and tenure) may be more willing to make extra effort for their organization (Tangirala and Ramanujam, 2008; Duan et al., 2017). Further, following prior studies (e.g., Bai et al., 2016; Liu and Li, 2018), leaders’ age, gender, and education, as team-level control variables, were also controlled.

## Results

### The measurement model

We first conduct confirmatory factor analyses using Mplus7.4 to examine the discriminant validity of our four main variables ethical leadership, psychological safety climate, moral self-efficacy, and constructive deviance. Table 1 shows that the four-factor model (χ^2^/*df* = 2.033, RMSEA = 0.058, SRMR = 0.0422, CFI = 0.945) has a better fit with the data than the alternative models, indicating that the discriminant validity of the four constructs was good.

**TABLE 1:** Results of confirmatory factor analyses.

Model	χ^2^	*df*	χ^2^/*df*	RMSEA	CFI	SRMR
Four-factor model: EL, PSC, MSE, CD	546.979	269	2.033	0.058	0.945	0.0422
Three- factor model: EL, PSC+MSE, CD	1201.993	272	4.419	0.105	0.817	0.1054
Two-factor model: EL+ PSC+MSE, CD	1726.965	274	6.303	0.130	0.713	0.1060
One- factor model: EL+ PSC+MSE+CD	1898.253	275	6.903	0.138	0.680	0.1047

EL, ethical leadership; PSC, psychological safety climate; MSE, employee moral self-efficacy; CD, employee constructive deviance; “+” requests the combination of factors.

### Common method variance checking

Following previous studies’ suggestions regarding checking common method bias (Podsakoff et al., 2003; Liang et al., 2021), the unmeasured latent method construct approach is used to analyze our collected data to further examine whether common method variance could bias our results. Specifically, we add the common method bias factor to the baseline model (i.e., the four-factor model) and contrast the fit indices of the two models. The fit indices of the five-factor model do not show a significant improvement (ΔSRMR = 0.0046, ΔRMSEA = 0.002, ΔCFI = 0.003), indicating that common method variance should not be a serious concern.

### Descriptive statistics

Descriptive statistics and correlations of all studied variables are presented in Table 2. Correlation analyses show that moral self-efficacy is positively associated with constructive deviance (*r* = 0.653, *p* < 0.01), and ethical leadership is positively associated with psychological safety climate (*r* = 0.674, *p* < 0.01).

**TABLE 2:** Correlation and descriptive statistics.

Variables	*M*	*SD*	1	2	3	4
**Individual level**						
1. Employees’ gender	1.63	0.485	−0.158**	0.760**	−0.032	0.653**
2. Employees’ age	2.57	0.607	−0.192**	−0.039	−0.012	
3. Employees’ tenure	2.89	0.885	−0.100	−0.022		
4. MSE	3.93	0.782	−0.079			
5. Constructive deviance	3.68	0.751				
**Team level**						
1. Leaders’ gender	1.15	0.355	−0.162**			
2. Leaders’ age	2.98	0.557				
3. Leaders’ education	3.28	0.551	0.266**	0.083	−0.031	0.674**
4. Ethical leadership	3.77	0.730	−0.089	−0.249**	−0.126*	
5. PSC	3.78	0.465	−0.258**	−0.024		

n = 313 individuals, N = 52 teams.

*p < 0.05, **p < 0.01.

### Hypothesis testing

Using Mplus7.4, we first examine the null model with no predictors to provide support for further multilevel analyses. The test results showed that the intragroup variance, intergroup variance, and ICC values of constructive deviance were 0.197, 0.348, and 0.644, respectively, supporting the use of the data in cross-level analysis.

#### Direct and mediating effects testing

As Model 2 of Table 3 shows, after including control variables, ethical leadership positively and statistically significantly predict employees’ constructive deviance (β = 0.727, *p* < 0.01), thus supporting Hypothesis 1. Then we test Hypothesis 2 and Hypothesis 3, that is, the mediating effects of moral self-efficacy and psychological safety climate, respectively. As shown in Model 3 of Table 3, after introducing ethical leadership and moral self-efficacy into the regression equation, the effect of ethical leadership on employee constructive deviance is still statistically significant, but the coefficient decreases from 0.727 (see Model2) to 0.517 (*p* < 0.01). With 20,000 Monte Carlo replications, the indirect effect of ethical leadership on constructive deviance through moral self-efficacy is statistically significant (estimate = 0.204, 95% CI = [0.144, 0.271], not containing zero), indicating that moral self-efficacy plays a partial mediating role. Thus, Hypothesis 2 was supported. As shown in Model 4 of Table 3, after adding the mediator variable for psychological safety climate into the model, the coefficient of ethical leadership’s influence on employee constructive deviance remains statistically significant, but decreases to 0.395 (*p* < 0.01). Employing a Monte Carlo simulation procedure, we also find a significant indirect effect of ethical leadership on constructive deviance *via* psychological safety climate (estimate = 0.334, 95% CI = [0.188, 0.507], not containing zero). Therefore, these results identify a mediating effect of psychological safety climate, and Hypothesis 3 is supported.

**TABLE 3:** Hypothesis test statistics.

Variables	Constructive deviance			
	Model 1	Model 2	Model 3	Model 4
Employees’ gender	−0.126	−0.110	−0.052	−0.093
Employees’ age	−0.108	−0.128	−0.067	−0.139
Employees’ tenure	0.034	0.048	0.037	0.044
Leaders’ gender	−0.361*	−0.133	−0.208	0.044
Leaders’ age	−0.132	0.123	0.114	0.062
Leaders’ education	0.042	0.003	−0.010	0.045
Ethical leadership		0.727**	0.517**	0.395**
PSC				0.768**
MSE			0.425**	
Intragroup variance	0.197	0.197	0.117	0.198
Intergroup variance	0.348	0.085	0.078	0.022

*p < 0.05, **p < 0.01.

#### Moderating effect testing

**FIGURE 2:**
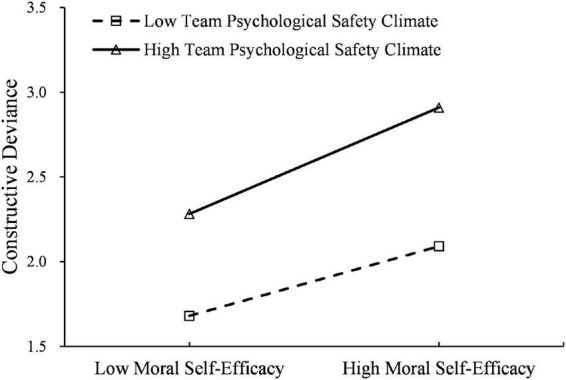
The moderating effect of team psychological safety climate on the relationship between moral self-efficacy and constructive deviance.

Hypothesis 4 predicts that psychological safety climate moderates the impact of employee moral self-efficacy on constructive deviance. Multilevel modeling results show that psychological safety climate has a positive influence on the random slope between employee moral self-efficacy and constructive deviance (β = 0.190, *p* < 0.05), indicating that cross-level interaction exists. Following Aiken and West’s (1991) procedures, we further plot this interaction at different levels of psychological safety climate. As shown in Figure 2, the relationship between employee moral self-efficacy and constructive deviance is stronger for a high level of psychological safety climate than for a low level of psychological safety climate. Therefore, Hypothesis 4 is supported.

#### Moderated mediating effect testing

Hypothesis 5 predicts the moderating role of psychological safety climate in the indirect effect of ethical leadership on constructive deviance. To test the hypothesis, we use Mplus7.4 and R software to identify whether the indirect effect is statistically significant across different levels of psychological safety climate. The results show that for higher ( + 1 *SD*) levels of psychological safety climate, this indirect effect is stronger (β = 0.210, *p* < 0.01); for lower (−1 *SD*) levels of psychological safety climate, this indirect effect is weaker and not statistically significant (β = 0.143, *p* > 0.01). However, the value of the between-group difference was not statistically significant (β = 0.067, 95 % CI = [-0.022, 0.157], including zero), and so does not indicate that the positive indirect effect of ethical leadership on constructive deviance *via* moral self-efficacy is moderated by the psychological safety climate. Therefore, Hypothesis 5 is not supported.

## Discussion

Based on social learning and uncertainty reduction theories, this study investigates whether and how team-level ethical leadership influences employees’ constructive deviance. We find a cross-level effect of ethical leadership on constructive deviance, wherein psychological safety climate and employee moral self-efficacy are mediating mechanisms. Moreover, psychological safety climate moderates the relationship between employee moral self-efficacy and constructive deviance, although it does not have a statistically significant moderating role in the indirect relationship between ethical leadership and constructive deviance that is mediated by employee moral self-efficacy.

### Theoretical implications

First, our findings reveal a cross-level positive effect of ethical leadership on employees’ constructive deviance. Therefore, our empirical analysis enhances our understanding of the outcomes of team ethical leadership, and provides evidence for the impact of ethical leadership on employees’ behaviors at the team-level in China. In addition, prior studies mainly focus on the effect of leadership style on employees’ constructive deviance at the individual level, and less attention has been given to this relationship at the team level, particularly in a Chinese context. Vadera et al. (2013) have called for more studies on the facilitators and inhibitors of constructive deviance using cross-level analysis. Wang (2022) has also stressed the imperative of exploring team-level antecedents of constructive deviance. This study responds to these calls by exploring the influence of team-level contextual factors (i.e., ethical leadership) on employees’ constructive deviance.

Second, most prior studies rely on theoretical perspectives such as social identity theory or social exchange theory to explain how leadership style influences employees’ constructive deviance. Our study contributes to the existing research from two perspectives. First, by integrating social learning and uncertainty reduction theories, we provide additional theoretical lenses to uncover the influencing mechanisms of leadership style on employees’ constructive deviance. Second, we identify psychological safety climate and moral self-efficacy as two important mediating mechanisms that link ethical leadership and employees’ constructive deviance. Our study therefore offers a more complete understanding of how team-level ethical leadership influences employees’ pro-organizational behavior (e.g., constructive deviance) by introducing different mediating mechanisms into our cross-level process model.

Third, this study reveals the key role of external contextual factors as boundary conditions in the link between leadership style and constructive deviance by demonstrating the moderating effect of psychological safety climate. Previous studies on boundary conditions of constructive deviance focus on individual traits, such as employees’ traditionality or positive personality, and studies on the impact of external contextual factors as moderators of constructive deviance are scarce. Our study enhances our understanding of the boundary effects of external contextual factors on constructive deviance by empirically examining the moderating role of team psychological safety climate in employees’ moral self-efficacy-constructive deviance relation. Hence, we more completely discuss when and why employees are willing to engage in constructive deviance.

Finally, psychological safety climate is found to moderate the association between employee moral self-efficacy and constructive deviance. However, our findings did not support the moderated mediation effect, which suggests that other plausible intervening variables may exist. For example, *guanxi*, a type of norms of interaction grounded in the Chinese Confucian ethical system, profoundly influences Chinese employees’ behavior (Chen and Bedford, 2022). For Chinese employees, establishment and maintenance of relationships with their supervisors are not limited to work, but also occur in a large amounts of non-work-related social activities. Such personal relationships with supervisors even influence employees’ work behaviors (Ko et al., 2017). Ding et al. (2017) show that *guanxi orientation* may weaken the positive impact of employee intrinsic motivation on voluntary behavior intention because individuals with high *guanxi orientation* value relationship harmony and pay little attention to self-actualization. Given that constructive deviance involves potential interpersonal conflicts, *guanxi orientation* may negatively influence employee constructive deviance, and thus could statistically offset the positive effect of team psychological safety climate on employees’ constructive deviance.

### Practical implications

Our findings have several practical implications for organizations. First, we show that team-level ethical leadership plays a vital role in motivating constructive deviance among team members. Thus, ethical leadership behaviors should be rewarded and developed. Relevant measures include promoting leaders who possess high ethical standards, linking the reward and punishment system to assessment of ethical leadership behaviors, and developing ethical leadership training programs that cultivate team leaders’ ethical leadership abilities.

Second, given the positive influence of employee moral self-efficacy on constructive deviance, leaders should develop this positive psychological state in their employees. Leaders’ primary efforts may concentrate on raising employees’ confidence in their ability to participate in ethical behaviors. For example, leaders may develop employees’ knowledge and understanding of ethical decision making by involving them in moral discourse and discussing moral issues with them. Also, using case studies and role-taking are both effective ways to enhance employees’ moral efficacy. When employees’ moral efficacy has been developed, they may become more confident in their ability to successfully implement constructive deviance.

Finally, to create a climate of team psychological safety, leaders must consider potential interpersonal risks and uncertainty in the workplace. They should recognize that they can function as organizers and coordinators to guide employees to trust and respect each other and reduce employees’ concerns regarding potential interpersonal conflicts. By doing so, leaders can provide a psychologically safe climate for their subordinates, which in turn, helps to facilitate the emergence of constructive deviance in the team.

### Limitations and future research directions

Our research has some limitations that warrant consideration. First, although we obtain data from multiple sources, the problem of social desirability may still influence our research results. Specifically, when employees report the level of their supervisor’s ethical leadership, they may conceal their true perceptions out of consideration of social desirability (Loo and Thorpe, 2000; Spector, 2006; Feng et al., 2016). Therefore, in accordance with Feng et al. (2016), we encourage future research to use random experiments and larger longitudinal samples to alleviate contamination due to social desirability.

Second, the construct of ethical leadership is proposed and developed based on Western business ethics. As stressed by Resick et al. (2006), the content of ethical leadership is varies across culture.

Influenced by Chinese collectivistic culture, Chinese employees are more concerned about whether ethical leaders’ behaviors meet the highest moral standards than are employees who are influenced by individualistic culture (Resick et al., 2006; Zhu et al., 2019). This difference may lead employees in different cultures to have different perceptions and reactions to the behavioral manifestations of ethical leaders, which may influence their job outcomes (e.g., constructive deviance). In this study, we do not control for the impact of this cultural effect because our participants were all from similar cultural settings. However, considering the question of cultural differences, we hope that future research can replicate or extend our model in different cultural contexts, which will help to attain a more nuanced understanding of ethical leadership’s impact on employee behaviors.

Finally, although we examine two different mediating mechanisms (i.e., psychological safety climate and moral self-efficacy), other potential linkage mechanisms have not yet been fully explored. For example, moral ownership is defined as a sense of responsibility that individuals feel for themselves, others, and organizational ethical actions. Ogunfowora et al. (2021) demonstrate that moral ownership encourages employees to conduct morally courageous behaviors. Likewise, intrinsic motivation is an individual’s vital motivational force, and it may cognitively stimulate employees’ constructive deviance. These possibilities indicate that other linkage mechanisms may also influence the relationship between ethical leadership and employee constructive deviance. Therefore, future studies may continue investigating other potential mechanisms and further enhance our understanding of how ethical leadership influences employees’ constructive deviance.

## Data availability statement

The raw data supporting the conclusions of this article will be made available by the authors, without undue reservation.

## Ethics statement

Ethical approval was not provided for this study on human participants because ethical review and approval was not required for the study on human participants in accordance with the local legislation and institutional requirements. Written informed consent for participation was not required for this study in accordance with the national legislation and the institutional requirements.

## Author contributions

LS developed the conception and design of this study and drafted the manuscript. LY conducted data collection and analysis and critically revised the research manuscript. Both authors contributed to the article and approved the submitted version.

## Appendix

### Questionnaire items

**Ethical leadership** (Brown et al., 2005).

1. Our team leader listens to what employees have to say.

2. Our team leader disciplines employees who violate ethical standards.

3. Our team leader conducts his/her personal life in an ethical manner.

4. Our team leader has the best interests of employees in mind.

5. Our team leader makes fair and balanced decisions.

6. Our team leader can be trusted.

7. Our team leader discusses business ethics or values with employees.

8. Our team leader sets an example of how to do things the right way in terms of ethics.

9. Our team leader defines success not just by results but also the way that they are obtained.

10. When making decisions, our team leader asks “what is the right thing to do?”.

**Moral self-efficacy** (Hannah and Avolio, 2010)

This 5-item moral efficacy items are available at https://www.mindgarden.com/121-moral-potency-questionnaire.

**Team psychological safety climate** (Liang et al., 2012)

1. In my work unit, I can express my true feelings regarding my job.

2. In my work unit, I can freely express my thoughts.

3. In my work unit, expressing your true feelings is welcomed.

4. Nobody in my unit will pick on me even if I have different opinions.

5. I’m worried that expressing true thoughts in my workplace would do harm to myself (reverse-coded).

**Constructive deviance** (Galperin, 2012)

1. My subordinate seeks to bend or break the rules in order to perform their job.

2. My subordinate violates company procedures in order to solve a problem.

3. My subordinate departs from organizational procedures to solve a customer’s problem.

4. My subordinate bends a rule to satisfy a customer’s needs.

5. My subordinate departs from dysfunctional organizational policies or procedures to solve a problem.

6. My subordinate reports a wrong-doing to co-workers to bring about a positive organizational change.

7. My subordinate does not follow my orders in order to improve work procedures.

8. My subordinate disagrees with others in their work group in order to improve the current work procedures.

9. My subordinate disobeys my instructions to perform more efficiently.

10. My subordinate reports a wrong-doing to another person in the company to bring about a positive organizational change.
